# Targeting Triple-Negative Breast Cancer: A Special Focus on Phototherapy and Nanomaterials

**DOI:** 10.3390/molecules31030511

**Published:** 2026-02-02

**Authors:** Ricardo Pereira, João M. P. Coelho, Maria Manuela Gaspar, Catarina Pinto Reis

**Affiliations:** 1 Research Institute for Medicines (iMed.ULisboa), Faculty of Pharmacy, Universidade de Lisboa, Av. Professor Gama Pinto, 1649-003 Lisboa, Portugal; pereiraricardo@edu.ulisboa.pt; 2 Instituto de Biofísica e Engenharia Biomédica (IBEB), Faculdade de Ciências, Universidade de Lisboa, Campo Grande, 1749-016 Lisboa, Portugal

**Keywords:** triple-negative breast cancer, light-based therapy, photothermal therapy, gold nanoparticles

## Abstract

Triple-negative breast cancer (TNBC) is an aggressive subtype of breast cancer lacking estrogen, progesterone, and HER2 receptors. This characteristic limits the effectiveness of hormonal and targeted therapies, and despite advances in chemotherapy (ChT), radiotherapy (RT), surgery, targeted therapy (TT) and immunotherapy (IT), clinical outcomes remain poor, highlighting an urgent need for new therapeutic strategies. The development of advanced nanotechnology-based strategies has opened new avenues for the diagnosis and therapy of TNBC. This review focuses on photothermal therapy (PTT) combined with nanotechnology-based strategies. PTT constitutes an emerging modality for oncological treatment that leverages light irradiation, mostly in the near-infrared (NIR) spectral region, to induce the localized thermal ablation of malignant tissues. When combined with gold nanoparticles (AuNPs), PTT is significantly potentiated. AuNPs have distinctive optical and physicochemical characteristics, rendering them highly effective as multifunctional nanoplatforms. Upon irradiation, AuNPs act as efficient photothermal agents, inducing localized hyperthermia. This thermal effect disrupts cellular homeostasis and initiates a cascade of cell death pathways, including apoptosis and necrosis, culminating in tumor regression. This review describes the latest therapeutic advances of PTT and AuNPs. As this innovative approach progresses toward clinical application, future studies and trials will be crucial in determining its potential for TNBC management and improving patient outcomes.

## 1. Introduction to Triple-Negative Breast Cancer 

### 1.1. Understanding Triple-Negative Breast Cancer

Breast cancer (BC) accounts for the second highest incidence among all cancers, by gender and age, and ranks fourth in mortality [1]. Triple-negative breast cancer (TNBC) is defined by the absence of an estrogen receptor (ER), progesterone receptor (PR) and human epidermal growth factor receptor-2 (HER-2). TNBC specifically accounts for 10 to 20% of all BC diagnoses. It is highly invasive, commonly diagnosed in younger women and tends to relapse faster [2,3]. It has an increased risk of metastasis in the lungs, liver and central nervous system. The five-year mortality rate approaches 40%, rising to nearly 75% in cases of early recurrence. When metastasis occurs, the five-year survival may fall below 11% [4,5,6]. Therefore, TNBC is usually considered a moderate- to high-risk malignancy [7].

Based on mRNA expression profiles, TNBC’s classification was initially divided into the following six molecular subtypes: two basal-like, mesenchymal, mesenchymal stem-like, immunomodulatory, and luminal androgen receptor (LAR) [8,9,10]. Subsequent refinements identified the following four main clusters (TNBCtype-4): basal-like 1 and 2, mesenchymal, and LAR, with the basal-like subtype representing approximately 70% of TNBC cases [3,9,10,11,12]. These tumors frequently harbor BRCA1/2 mutations, show elevated proliferation rates, and overexpress genes related to DNA repair, making them susceptible to Poly ADP-Ribose Polymerase-1 (PARP) inhibition [10,13,14]. Mesenchymal type is characterized by the upregulation of JAK and PI3K, whereas immunomodulatory TNBCs display high immune checkpoint expression, potentially responsive to immunotherapy (IT). Lastly, the LAR subtype, characterized by androgen receptor dependence, may benefit from receptor antagonists [10,11,12,14]. Burstein et al. refined this latter idea of classification based on gene expression into the following two types: basal-like immune-suppressed and basal-like immune-activated, which are both distinguished by the absence or presence of tumor-infiltrating lymphocytes, respectively. The mesenchymal and luminal androgen receptor types were kept [9,15].

The tumor microenvironment (TME) is defined as a biosystem where tumoral cells coexist with healthy cells, such as fibroblasts, immune cells, and endothelial cells. Any changes that occur in the TME might lead to tumor progression, increasing the probability of metastasis occurring [16,17]. The TME presents a heterogeneous immune environment, with cells such as Treg cells, which contribute to the immunosuppressive response by expressing Cytotoxic T-Lymphocyte Antigen-4 (CTLA-4), downregulating T-cell activity and thereby blunting anti-tumor activities.

Another concern is hypoxia, which is generated due to cancer-associated fibroblasts producing extracellular matrix products and therefore increasing tension in that same matrix. From then on, vascular endothelial growth factor (VEGF) and other pro-angiogenic factors are produced by both tumors and fibroblasts, leading to neoangiogenesis. The new blood vessels, which are abnormal, not only enhance the blood supply to the malignant cells but also provide a route that may facilitate metastasis. Ongoing research seeks to unify the presented molecular classifications through integrative analyses combining genomic, transcriptomic, and microRNA profiles, aiming to improve TNBC stratification and guide personalized therapeutic approaches.

The aim of this review is to provide an overview of the conventional treatments of TNBC, such as surgery, chemotherapy (ChT), radiotherapy (RT), targeted therapy (TT), and IT, but also new advances and potential strategies, including light-based therapies. This review compiles articles obtained through a search on Web of Science and PubChem databases using the keywords “Triple-Negative Breast Cancer”, “Immunotherapy”, “Targeted Therapy”, “Nanotherapy”, and “Photothermal Therapy” (AND/OR). Only articles published in English were selected from indexed journals, with preference given to those published in the last 10 years. Conference proceedings were excluded.

### 1.2. Clinically Available Therapies for TNBC

TNBC lacks common molecular targets, not responding to hormone therapy or intervention, such as lumpectomy or mastectomy, which is often the first step for localized tumors. This is typically followed by RT to reduce the risk of local recurrence. Systemic ChT, however, plays a crucial role in both early and advanced TNBC stages due to the high responsiveness to cytotoxic agents. Standard chemotherapeutic regimens involve agents such as anthracyclines, taxanes, and platinum-based compounds. Despite its initial sensitivity to ChT, TNBC is associated with a higher risk of early relapses and metastasis compared to other BC subtypes. Therefore, the management of TNBC using conventional therapies is complex and often necessitates a multidisciplinary approach. The following sections describe the therapeutic options in clinical use as well as challenges associated with them; personalized treatments are also mentioned, focusing not only on their advantages but also on the importance of investigating new and alternative strategies, such as BRCA-targeted treatments, making the management of this malignancy highly challenging. For instance, tamoxifen, frequently used in hormone therapies, and trastuzumab, which targets HER2, are ineffective. Therefore, conventional ChT drugs are chosen in order to eliminate or, at least, slow tumor progression. The mainstay of conventional TNBC therapies includes surgery, RT, and ChT.

Surgery is always the first option in the case of clear margins, when performing mastectomy, lumpectomy, and breast conservative surgery, since it can provide lower adverse effects when compared to ChT and/or RT [18]. Each of the surgery methods presented does not have any benefit over the others either in prognosis or in the risk of recurrence; therefore, breast-conserving surgery is usually the most preferred option, since it preserves aesthetics, which is highly valued in women patients [19]. Surgery has advanced throughout the years, going from radical mastectomy, which left women with deformations, to surgeries where oncology and plastic surgery align in order to rebuild women’s breasts. Aside from that, sentinel lymph node biopsies are taken to ensure that no residual tumor cells have migrated through the lymphatic system, causing metastasis in distant organs [18].

RT is usually applied as adjuvant therapy since it eliminates any residual cancer cells that remain in the breast or the lymph nodes, being applied to patients who undergo breast-conserving surgery [18].

ChT has been shifting towards neoadjuvant therapy, which occurs before surgery, since TNBC is usually chemo-sensitive in the early stages. Additionally, it can be used as adjuvant therapy, serving the purpose of eliminating any residual TNBC left after a surgical procedure, hence reducing any risk of recurrence, deemed to be high in this subtype [14,18].

Doxorubicin is one of the main standard drugs of anthracyclines and is usually used in combination with taxanes. However, doxorubicin is known to be cardiotoxic, which requires the assessment of this risk when selecting a protocol regimen for patients. The second group of drugs are taxanes, known as microtubule targeting agents. When combined with anthracyclines, taxanes contribute to a decrease in the mortality rate by almost 20% [12,14,20]. The pathological complete response is around 35% in TNBC, whereas in BC it reaches 76% [20,21]. More recently, epothilones have become novel types of microtubule-targeting agents, which are being tested to surpass the resistance mechanisms of taxane therapies [12].

However, chemoresistance is a common situation in TNBC, which tends to increase the risk of metastatic cancer [19]. The tumor cells develop strategies that lead to maintaining their viability, such as reduced DNA repair enzyme, topoisomerase II, as a response to treatment with anthracyclines, and an increase in the expression of β-tubulin III, which promotes resistance to taxanes. Furthermore, TNBC cells have ATP-binding cassette transporters, which encourage the exclusion of drugs by transmembrane efflux [20].

In clinical practice, Table 1 summarizes the current ESMO (2024–2025) guidelines, which outline various treatment options besides ChT and surgery [22]. Herein, targeted therapy (TT) and immunotherapy (IT) can also be considered as potential strategies, either as primary treatments or as components of combination regimens.

The first advancement in targeted therapy (TT) was researching any possible receptors that could lead to targeting in this subtype of BC. Hence, molecular therapies were developed to modify the expression and response of receptors, proteins, and hormones, or even to interfere with DNA replication and repair mechanisms. The most notable classes of drugs within TT are PARP inhibitors, followed by epidermal growth factor receptor (EGFR) and vascular endothelial growth factor receptor (VEGFR) inhibitors.

PARP inhibitors inhibit poly ADP-ribose polymerase (PARP), an enzyme that helps repair single-strand breaks in DNA. Additionally, BRCA1 and BRCA2 are tumor suppressor genes that encode proteins that are responsible for repairing double-strand breaks through homologous recombination [23]. The purpose of using PARP inhibitors is to increase the number of errors in single-stranded DNA, allied to BRCA mutations that increase the failing of double-stranded DNA repair, culminating in cell death. Olaparib and Talazoparib are examples of PARP inhibitors and are used to treat patients with HER2- and TNBC with BRCA mutations. Unfortunately, not even 20% of these cancers have the mutation, so it is not a common treatment [24].

Additionally, EGFR is present in 30 to 60% of TNBC, which was found to be promising as a target of inhibitory drugs [25]. Gefitinib, part of EGFR inhibitors, blocks the proliferation of BC cells, and it is usually combined with other agents, namely docetaxel and carboplatin, which might synergistically increase the tumor progression [26]. Even though many are being trialed on, to the best of our knowledge, the only approved one is Lapatinib and as part of a combination therapy. The in vivo models had greater results than clinical trials, due to higher tumor heterogeneity in receptor expression, causing drug resistance. One of these is AXL, part of the receptor tyrosine kinase family, which is also overexpressed in TNBC and enables lymphovascular mechanisms, increasing the invasiveness and survival of tumors [27].

On the other hand, for tumor neo-angiogenesis, it is important to cut off the blood and nutrient supply by targeting VEGFR, which is expressed at higher levels compared to non-TNBC. The most common drug used is bevacizumab, an antibody that blocks VEGF binding to VEGFR. Additionally, VEGFR inhibitors are being researched, such as sorafenib, although clinical trials appear contradictory due to toxicity issues and drug resistance [26,28].

Immunotherapy (IT) is based on altering or enhancing the immune system in order to increase the immune response against the tumor cells. The presence of tumor-infiltrating lymphocytes increases the responsiveness to treatment with immunomodulatory drugs. IT has already been used in many other solid tumors, such as lungs and melanoma, and has prolonged the life of those patients [13,29].

The main IT drugs used are immune checkpoint inhibitors (ICIs), which suppress the immune response pathways, such as programmed death ligand-1 (PD-L1), PD-1 interaction and CTLA-4, therefore increasing the targeting of cancer cells [13,30]. PD-1 is an immune checkpoint receptor that binds to PD-L1 in tumor tissues, inhibiting the T-cell response against the cancer. The main examples of drugs from this class are pembrolizumab, nivolumab, and ipilimumab. The former ones target the PD-1 receptor, while the latter inhibits CTLA-4 [14,29]. Despite metastatic disease, prior chemotherapy, and PD-L1 positivity, pembrolizumab monotherapy showed promising activity in early trials, including the KEYNOTE-012 study [30,31]. In addition to the early monotherapy data, clinical trial KEYNOTE-522 [32] demonstrated substantial efficacy for pembrolizumab in combination with ChT, which underpinned regulatory authorization in TNBC. However, results from several other KEYNOTE trials were not as positive; thus, its use as monotherapy in early-stage disease has not been adopted as first-line [30,32,33].

Aside from PD-1, and given its role in immune suppression, CTLA-4 has become a target for IT such as ipilimumab. Blocking CTLA-4 with monoclonal antibodies can restore DC function, enhance T-cell activation, and directly inhibit tumor cell proliferation [34]. Furthermore, combining CTLA-4 blockade with other treatments, such as MUC1 mRNA nano-vaccines, has shown promise in enhancing anti-tumor CTL activity, since the MUC1 mRNA increases the CTLA-4 expression. The antibody used resulted in modulating the tumor microenvironment in TNBC models and potentiating the anti-tumor response [35].

**Figure 1:**
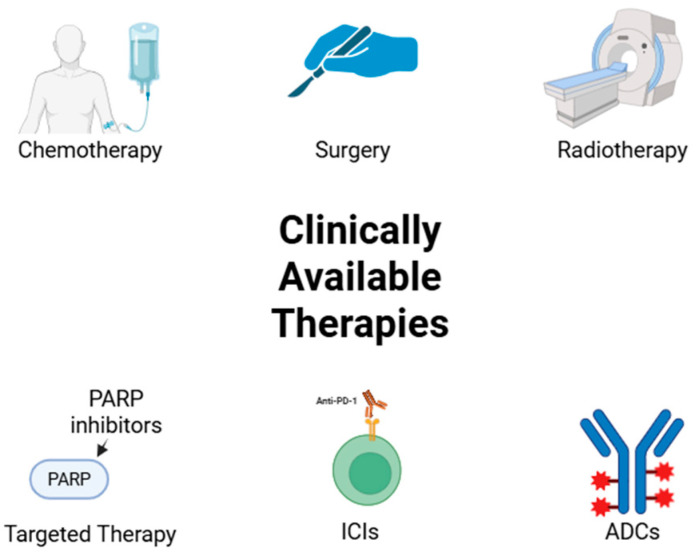
Clinically available therapies for TNBC. ADC: antibody-drug conjugates; ICIs: immune checkpoint inhibitors; PARP: poly ADP-ribose polymerase; PD-1: programmed death receptor 1. Created with BioRender.com.

Additionally, antibody-drug conjugates (ADCs) are also a combinational therapy for TNBC, which is based on conjugating a monoclonal antibody with a cytotoxic drug. These ADCs have been tested in patients with solid and liquid tumors, which presented great efficacy and safety profiles. Examples from this strategy are glembatumumab vedotin (GV) and Sacituzumab govitecan (SG). SG is a third-generation ADC and is already approved by the FDA for treating pre-treated metastatic TNBC, which targets Trop-2, a glycoprotein [29,36]. The ASCENT trial compared SG to other treatments in advanced TNBC, leading to a rapid approval due to the increased overall response rate. Even though the approval is meant for metastatic TNBC pre-treated with two regimens, at least one for the advanced condition, this ADC has no adverse effects in the respiratory and cardiovascular setting, which is fairly common in other approved ADCs [36,37]. Figure 1 resumes the clinically established therapies for TNBC.

### 1.3. From Established to Emerging Therapeutic Strategies in TNBC

While TT and ICIs represent the current cornerstone of TNBC treatment, ongoing research continues to expand the therapeutic landscape toward more sophisticated and personalized approaches. The success of these clinical therapies has opened the door to exploring next-generation immunotherapeutic modalities, aiming not only to enhance tumor eradication but to also establish long-term immune protection and overcome mechanisms of resistance observed in clinical settings.

Cancer vaccines, oncolytic viruses, and Chimeric Antigen Receptor T-cells (CAR-T) are other options. Cancer vaccines increase immune response against tumors, not only by improving the elimination of tumor cells but also by building an immunity memory against cancer. These vaccines are now being made with the use of nanotechnology, such as nanoliposomes, phages, bacterial subunits, and viruses, to carry out nucleic acids and peptides preferentially [38,39]. In clinical trials, there is one cancer vaccine that is testing a lysovirus expressing CTLA-4, which will try to increase the immune response [26].

Oncolytic viruses constitute another frontier in immunotherapy. They are viruses that infect tumor cells with the intent of replicating, causing lysis. Subsequently, they spread in the tumor microenvironment and infect other tumor cells, and the cycle starts again. This is noted as a direct way of eliminating cancer cells. Alternatively, there is an indirect approach, where the oncolytic viruses “target” the immune system by improving its response against the cancer. This is performed by releasing tumor-associated antigens whenever lysis occurs, which activates antigen-presenting cells and causes a further increase in the activity of CD8+ T-cells [40].

CAR-T cell therapy in TNBC is currently under investigation and is not yet part of standard clinical practice. Although CAR-T therapy has shown remarkable success in hematological malignancies, its application in solid tumors such as TNBC remains challenging. CAR-T cell treatment is based on using T-cells that have the CAR receptors expressed, which bind to specific targets in tumors and further cause immune response activation [41]. The process of producing CAR-T cells is the following: (1) Patients have their blood taken, peripherally where T-cells are available, (2) the CAR gene is inserted and CAR-T cells are now developed to target tumors, (3) the same CAR-T cell is multiplied, and (4) they are injected into the same patient. A fifth-generation CAR-T has been developed, where the interdomains were augmented to target many signaling pathways, to further increase immune response against tumors, and also to increase T-cells’ presence in the tumor microenvironment [42]. This therapy also has setbacks; the heterogeneous nature of solid tumors contrasts with the hematological tumors, where many CAR-T target the B-cell CD19 [43]. Additionally, they might induce toxicity when they release cytokines, leading to neurotoxicity or cytokine release syndrome. Thus, very precise targeting must be researched in order to obtain the most out of this therapy [42].

### 1.4. Challenges to Overcome

One of the primary obstacles in treating TNBC with current therapies lies in the need for the tumor to exhibit particular biological features—such as mutations in the BRCA1 or BRCA2 genes or the presence of specific receptors—to ensure that the therapeutic agents can effectively target the cancer cells. This specificity limits the broad applicability of certain treatments across all TNBC patients [44]. Additionally, in the case of IT, its effectiveness is similarly constrained; if tumors lack a sufficient presence of tumor-infiltrating lymphocytes or develop genetic adaptations that suppress the immune response, then ICIs are unlikely to produce a therapeutic benefit [45,46]. This underscores the importance of biomarker testing as a routine component of clinical decision-making when considering IT, to identify those patients who are most likely to respond. As such, ongoing research is vital, not only to enhance the precision of existing treatments but also to incorporate emerging therapeutic strategies that may overcome these biological barriers and ultimately lead to improved outcomes for a broader range of TNBC patients.

**Figure 2:**
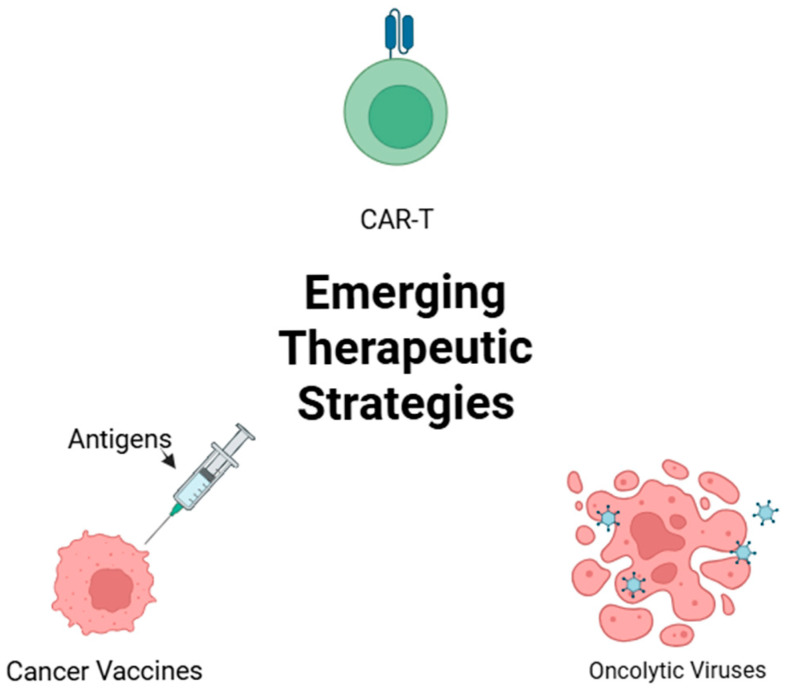
Emerging therapeutic strategies. CAR-T: Chimeric Antigen Receptor T-cells; ROS: reactive oxygen species. Created with BioRender.com.

Consequently, research now focuses on optimizing established approaches and combining them with innovative therapies to enhance patient outcomes; notably, light-based modalities have garnered increasing attention. Figure 2 illustrates emerging therapies whose clinical implementations are still discrete.

## 2. Light-Based Therapies

**Figure 3:**
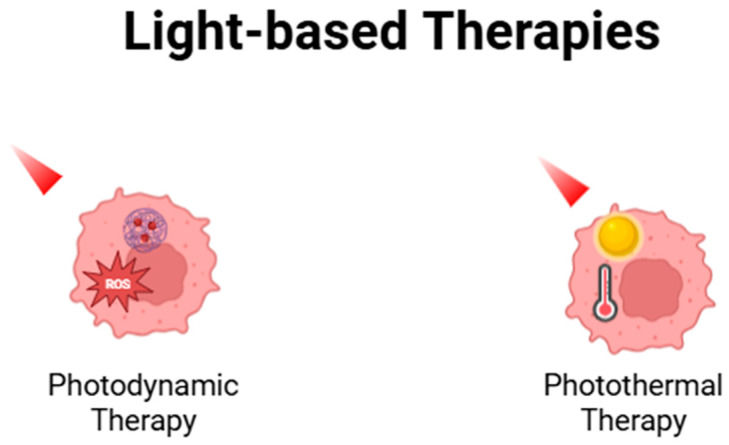
Light-based therapies. ROS: reactive oxygen species. Created with BioRender.com.

As an alternative, light-based therapies (Figure 3) include photodynamic therapy (PDT), photothermal therapy (PTT), and photoimmunotherapy (PIT), which use defined wavelengths of light to activate photosensitizers or other light-sensitive agents at the tumor site. This controlled activation produces spatially confined tumor cell killing, can prime antitumor immune responses, and limits collateral injury to surrounding healthy tissue. Their non-invasive nature, potential for repeatable application, and ability to be combined with other modalities—such as ChT or IT—make them promising candidates for managing TNBC [47].

### 2.1. Light as a Quantum Mediator of Energy

As a physical stimulus, light possesses unique properties such as non-invasiveness, spatiotemporal controllability, and spectral selectivity, making it an ideal trigger for confined therapeutic activation within target tissues.

Upon optical excitation, nanomaterials absorb photons and convert photonic energy into either thermal energy (PTT) or reactive oxygen species (ROS) via triplet-state photochemical transitions (PDT mechanism). This photo-energetic transduction is governed by the electronic structure of the PS through localized surface plasmon resonance (LSPR) in, for example, gold nanoparticles (AuNPs), generating subcellular hyperthermia and protein denaturation, or through Type II energy transfer in porphyrinic systems, producing highly oxidative singlet oxygen.

The spectral modularity of light enables synergistic integration with IT, ChT, or RD, enhancing immunogenic cell death and reprogramming of the tumor microenvironment without increasing systemic toxicity.

As a key component of next-generation photoactive nanomedicine, light serves as a quantum mediator of energy rather than just a physical trigger, transforming photonic information into a precisely controlled bioenergetic perturbation.

### 2.2. Light Sources

In light-based therapies, the choice of light source is a critical determinant of treatment efficacy, penetration depth, and selectivity toward malignant tissues. Commonly employed sources include continuous-wave or pulsed lasers, e.g., diode or Nd:YAG lasers, offering high coherence and tunable wavelengths that match the optical absorption bands of nanomaterials such as AuNPs or porphyrin PS [48]. Light-emitting diodes (LEDs) represent a cost-effective and versatile alternative for broad-area or superficial illumination. They emit over a wider spectral range and generate less localized heating than lasers, making them particularly suitable for dermatological applications, wound healing, and photobiomodulation therapy. However, regarding cancer therapy, lasers are the equipment of choice as a light source [49].

For PTT, near-infrared (NIR) light—typically with wavelengths within 650–1350 nm—is preferred due to its optimal tissue penetration and minimal scattering [50]. In photodynamic therapy (PDT), light sources are selected to match the absorption maxima of the photosensitizer, commonly between 630 and 690 nm for porphyrins and chlorins [49]. The most common technology used is diode lasers, as they present a cost-effective solution with a wide range of wavelengths and power available. Emerging modalities exploit ultrafast femtosecond lasers or upconversion NPs activated by multi-photon excitation, allowing subcellular precision with reduced collateral damage, but with higher cost and increased complexity [50].

Light-based technology also allows for the incorporation of flexible beam delivery. The most common fiber-optic systems enable the minimally invasive delivery of light to deep-seated tumors through catheters or endoscopic probes, maintaining precise spatial control. Superficially, besides direct irradiation (which can be sized through optical zoom systems or just by changing the distance to the targeted area), scanning systems can provide a more “tailored” irradiation [51,52].

### 2.3. Photodynamic Therapy

**Figure 4:**
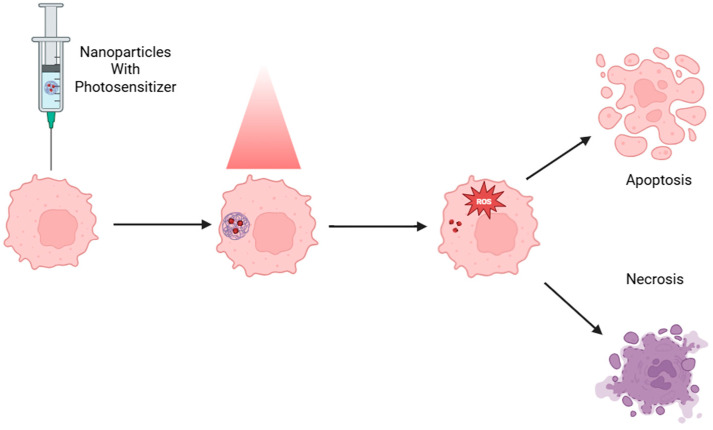
A schematic illustrating the PDT mechanism. ROS: reactive oxygen species. Created with BioRender.com.

Photodynamic therapy (PDT) implies the use of a non-toxic PS, which is selectively retained in tumor tissue and subsequently activated by light of a specific wavelength [53,54]. Upon irradiation, the PS produces ROS, which induces apoptosis in malignant cells (Figure 4) [53,54,55]. A significant advantage of PDT lies in its high degree of selectivity: the PS remains inactive and non-cytotoxic until exposed to light, allowing for localized treatment with minimal impact on surrounding healthy tissue. This spatial and temporal precision significantly reduces systemic toxicity—a common drawback of conventional ChT—and lowers the risk of drug resistance, as the therapeutic mechanism relies on immediate oxidative damage rather than sustained drug–target interactions [47]. Consequently, PDT offers a compelling alternative or adjunct to traditional therapies, particularly in tumors that are resistant to systemic agents or require minimally invasive intervention.

PS can be administered locally or intravenously and can be distributed to either healthy or cancerous cells, but the healthy tissues have natural lymphatic drainage, which is not the case with tumors. Thus, the PSs preferentially accumulate in tumors [56].

Another application of PDT is related to its immunomodulatory effects. ROS-mediated damage can lead to the release of tumor-associated antigens and danger-associated molecular patterns (DAMPs), thereby stimulating an immune response. This phenomenon has been shown to potentially convert “cold” tumors—those with low immune infiltration—into “hot” tumors, thereby increasing their susceptibility to immunotherapies. In the context of TNBC, which often presents with an immunosuppressive microenvironment, this immune-activating property is particularly significant.

Despite its potential, PDT faces several challenges, including limited light penetration into deep tissues, heterogeneous PS uptake, and the need for precisely controlled irradiation parameters. These issues are currently being addressed through advances in nanotechnology, which enable the encapsulation of PSs in biocompatible nanoparticles (NPs), facilitate targeted delivery, and utilize near-infrared light sources for enhanced tissue penetration.

As an example, one study was conducted with PDT and RT, either alone or in combination treatment [57]. Herein, a porphyrin PS was evaluated in vitro in MDA-MB-231 and 4T1 cells and in vivo in BALB/c female mice bearing 4T1 tumors established by cell inoculation. Regarding the in vitro findings, it was found that the combination of both treatments reduced cell survival when compared to RT alone, while PDT alone induced higher apoptosis, and the mitochondrial activity was decreased in PDT and combination groups when compared to RT. On the other hand, in vivo studies related the sequence of treatment more than the comparison between treatments. It was observed that PDT after RT performed better in clinical conditions, survival, and in decreasing metastasis than RT or PDT followed by RT. Another significant aspect is that it can reduce toxicity since it increases the effectiveness of RT at lower dosages and may even substitute ChT.

Unlike the previous study, many researchers have tried to establish and investigate how PDT would fare when used with systemic treatments, such as ChT and IT. Interesting results were observed when combining ChT drugs, such as cisplatin, PTX, and doxorubicin. One study researched 5-ALA as PS for PDT therapy and how it could impact cisplatin treatment. The study found that combining the therapy rather than sequential administration would lead to an increase in the early apoptosis process, at least in the MDA-MB-231 TNBC cells [58]. In addition to this, another research group tried to use chlorin conjugates as PS and evaluated the PDT combined with several common ChT agents in the BT-549 cell line. ChT drugs, such as fluorouracil, doxorubicin, and cisplatin, lead to a decrease in cell viability, whereas the treatment with ChT alone had worse results. The best combination found in this study was with PTX and/or fluorouracil combined with indium-chlorin-lipoic acid complex, which led to a cell inhibition of around 90% [59]. Despite these results being outstanding, there is a major factor that must be accounted for before progressing into other trials and studies: one is the fact that it was in vitro, so it does not account for systemic distribution throughout the healthy tissues, which ought to happen whenever ChT is used. On the other hand, this may lead to a decrease in chemoresistance and a decrease in the ChT dosage used to obtain the optimal results.

Another approach is to increase the immune response in the tumor area, which can be used with IT or with immunomodulatory drugs. Regarding the latter one, Banerjee et al. tested the combination of PDT with verteporfin as PS and 5-aza-2′-deoxycytidine, which is a methylation agent and is intended to increase the immune response. This combination is used to increase cytotoxicity and enhance the recognition of the tumor to enable the immune system to fight against it. A decrease in the viability of the tumor was found, with the enhancement of T-cell population activation and the downregulation of DNA methyltransferase and Yes-associated protein, which are responsible for tumor progression and further metastasis. The only setback of the study is TNBC as a fast progression cancer, which led to early sacrifices; thus, no long-term effects were assessed [60].

Liang and co-workers used nanoplatforms by adding PD-1/PD-L1 inhibitors BMS-202 to a metal-organic framework, where the objective was to integrate PDT into IT. An increase in apoptosis and necrosis in the tumor was observed, and more memory T-cells were present in mice, suggesting a decrease in the possibility of recurrence [61]. These studies suggest that PDT can be implemented in many other treatments that are already approved, creating chances of reducing ChT dosages and improving the IT response in TNBC, even though this could only be further assessed through in vivo studies with larger animals or clinical trials.

### 2.4. Photothermal Therapy

PTT has garnered considerable interest in recent years as a precise and minimally invasive treatment modality for cancer, and it is not significantly affected by the tumor microenvironment, in contrast to PDT [62,63]. Conventional PTT relies on a high thermal dose—commonly >50 °C for minutes—to induce coagulative necrosis and tumor ablation. This method can induce inflammation and also damage adjacent healthy tissues [64]. In contrast, mild-PTT (or mild-temperature photothermal therapy) employs sub-ablative temperatures (~42–45 °C) to sensitize tumors and stimulate immune responses, proving most effective as an adjuvant alongside ChT, IT, or PDT.

To improve the outcomes, PTT should operate through the use of photothermal agents (PTAs) such as NPs [64,65,66]. Some NPs, like AuNPs, possess the unique capability to have higher absorption on specific wavelengths of light and convert them into thermal energy [67,68,69]. Through plasmonic tuning, AuNP architectures (nanoflowers, nanorods, nanocages, nanopyramids, nanoshells) achieve NIR-range absorption and thus potentiate their effect.

**Figure 5:**
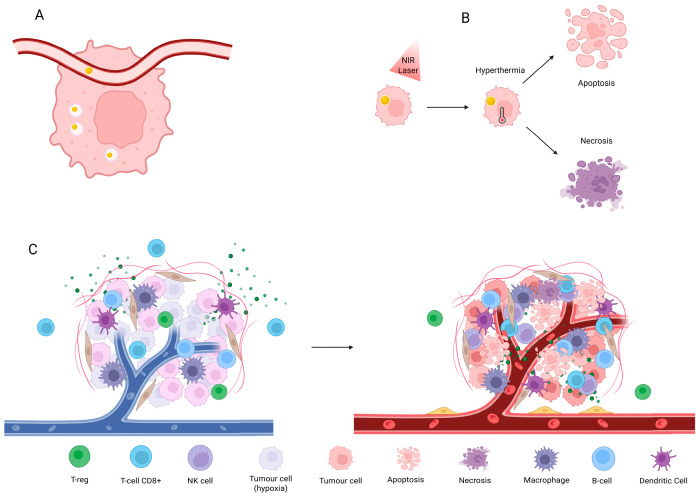
A schematic illustration of the PTT mechanism. (**A**) AuNPs injected IV, and tumor accumulation. (**B**) PTT mechanism. (**C**) TME modulation before and after PTT. NIR: near-infrared; NK cell: natural-killer cell. Created with BioRender.com.

PTT is performed in a tissue-transparent spectral window [70,71]. This increased localized heat generation induces hyperthermia within the tumor microenvironment, leading to cellular stress, protein denaturation, membrane disruption, and ultimately, tumor cell death (Figure 5).

The main advantages of these NPs are their physical properties, such as optical, thermal, magnetic, and electrical properties, which promote mechanisms of action against cancer cells. Their shape can be modified in order to have better outcomes or applications such as the delivery of some drugs [53]. The following Table 2 depicts the main properties of AuNPs and how they can impact the PTT efficiency in TNBC.

The potential mechanisms of action of AuNPs consist of (1) the generation of reactive oxygen species, which is the main one when using PDT, (2) heat generation, due to their thermal properties that make them suitable to be used in PTT, and lastly, (3) targeted delivery and intrinsic anti-cancer activity, which is based on functionalizing them by binding with molecules on their surface, or even as nanocarriers, which leads to an increase in specifically targeting the tumor site and avoiding any side effects within the body.

Consequently, AuNPs have been extensively studied for diagnostic and therapeutic purposes [76,77]. During the product development, it is important to ensure the safety and efficacy of the AuNPs. In TNBC research, the safety and efficacy of PTT using AuNP systems were evaluated in commercialized cell lines (2D cultures) and also in 4T1 BC cell lines transplanted in mice. As far as it is concerned, this cell line (4T1) is the one that shares the most similarity to a human TNBC [78,79]. The AuNPs and NIR laser irradiation alone were unable to affect the cell’s viability remarkably. However, when used in combination, cell death is significantly observed (above 70% in 4T1 cells). Regarding in vivo results, the combination treatment resulted in enhanced tumoral necrosis, where no difference was noted when using core and HAOA-coated formulations, apart from the protective effect in healthy cells.

A major factor in nanoparticles is the enhanced permeability and retention (EPR) effect [80]. It was conventionally thought that NPs would accumulate in tumors due to having dimensions that would evade renal clearance. Hyperthermia, surface modifications with specific ligands and active targeting all contribute to an increase in the EPR effect.

To enhance specificity and safety, PTT typically employs light in the first near-infrared (NIR) optical window—between 700 and 900 nm—where endogenous chromophores—principally oxy/deoxy-haemoglobin and melanin—exhibit relatively low absorption and water absorption is modest [55]. This window enables deeper tissue penetration and reduces off-target heating; when paired with tumor-localized PTAs, it allows precise tumor heating while preserving surrounding tissue. Accordingly, PTT holds strong promise as a stand-alone or adjunct modality in oncology, including hard-to-treat subtypes such as TNBC.

While PTT offers promising advantages in localized tumor ablation, its efficacy can be significantly compromised in the absence of actionable molecular targets and highly metastatic cancers such as TNBC. As previously described, TNBC is characterized by the absence of ER, PR, and HER2 receptors, which limits the effectiveness of receptor-mediated targeting strategies commonly used in NP-based therapies. When administered intravenously, non-targeted NPs may accumulate non-specifically, resulting in suboptimal therapeutic outcomes and increased risk of off-target effects. To address this limitation, modifications to the NP surface or formulation are necessary to enhance tumor selectivity and cellular uptake. A notable approach was demonstrated by Xu et al., who employed puerarin-based NPs (nanoPue) to transiently increase the porosity of tumor plasma membranes, thereby facilitating the infiltration of PTAs into tumor cells [81].

In addition, gold-core/silica-shell NPs embedding a water-soluble iridium(III) complex as a PS and luminescent probe were efficiently decorated with amino-terminated EGFR (CL4) and PDGFRβ (Gint4.T) aptamers. The targeting specificity and the synergistic PDT and PTT effects were assessed on different human cell types, including the mesenchymal subtype of TNBC [82]. In particular, the presence of the aptamers as active targeting ligands ensures a selective addressing, and the external stimuli-mediated triggering of the nanosystem provides spatio-temporal control of the cytotoxic action for a highly selective multimodal cancer treatment approach [83].

Another approach is to use gene therapy, where the NP, in this case a nanorod, is part of a nanosystem where it is embedded in a silica nanosphere [84]. This research group developed a system where a nanosphere of silica could carry SiRNAs and also nanorods, tackling the therapy from the following two perspectives: (1) the SiRNAs are widely diffused in the cytoplasm in order to silence overexpressed oncogenes, and (2) the silica nanospheres are irradiated with a laser to induce heat through the gold nanorods. Another group produced gold nanostars and attached siRNA that led to the downregulation of HSP72, a heat shock protein, and further increased the PTT efficiency [85].

Besides gold, a research group developed zirconium NPs using RT and PTT [76]. They coated the zirconium NPs with bovine serum albumin and attached folic acid to enhance their biocompatibility and targeting to TNBC cells, which often overexpress folate receptors. The results suppressed the progression of TNBC in a mouse model.

Apart from zirconium, copper can be considered as an alternative [86]. Platelet membrane-coated Cu_9_S_8_–SNAP NPs have demonstrated enhanced tumor accumulation via biomimetic targeting. The Cu_9_S_8_ core enables PTT and chemodynamic therapy (CDT), whereas the S-nitroso-N-acetylpenicillamine (SNAP) moiety functions as a nitric oxide (NO) donor, enabling gas therapy. Upon irradiation with NIR-II, the nanosystem induces localized hyperthermia, enhances ROS generation through metal-mediated reactions, and triggers controlled NO release, leading to increased tumor cytotoxicity. This multifunctional platform integrates PTT, CDT, and gas therapy, representing a promising combinatorial strategy for cancer treatment. Table 3 presents different nanomaterials used for the synthesis of nanoparticles in PTT.

Finally, PTAs can also be organic, like small molecules, such as anthocyanin and indocyanine green, also used in PDT, or polymers that can absorb light in the NIR range, where polydopamine, polyaniline, and polypyrrole are examples [87,88]. Polydopamine nanoparticles (NPs) loaded with olaparib represent an example of organic PTAs designed to target TNBC harboring BRCA mutations. In BALB/c nude mice bearing a BRCA1-mutated breast cancer cell line [89], treatment with a combination of PTT and olaparib-loaded NPs resulted in a tumor volume reduction exceeding 50% compared with either PTT or olaparib monotherapy. These findings highlight the potential of combinatorial photothermal–chemotherapeutic strategies to enhance the efficacy of established treatments, potentially reducing required drug doses and associated costs, while improving therapeutic outcomes.

**Table 3:** Nanomaterials used for the synthesis of NPs in Phototherapy.

Material	Photothermal Conversion Efficiency (PCE)	NIR Wavelength	Advantages	Limitations	Reference
Gold	Depends primarily on shape and polydispersity (almost 99%)	550 to 1100 nm	Leading research metal Tunable modifications in size, morphology and surface ligands attachments Low toxicity	Lower absolute heat (spheres) High sensitivity to polydispersity (rods) Complex synthesis (stars) Aggregation	[68,73,74,75]
Silver	50–80%	970 nm	Tunable surface plasma resonance Antimicrobial activity Reduced cost	Reduced penetration depth Leaching toxicity and risk to healthy cells during the laser irradiation Instability	[90]
Carbon Nanotubes	30–50%	400 to 1100 nm	Broad NIR absorption Good penetration depth Intratumoral injection in PTT-ChT with good results	Moderate PCE IV injection diminishes their impact Toxicity of the compounds	[91]
Copper	30–60%	800–1300 nm Preferentially at 980 nm for CuS NPs	Penetration depth enhanced in NIR-II window Lower cost Biocompatibility Combination with chemodynamic therapy	Moderate PCE when used with polydopamine coating Toxicity at 980 nm in healthy tissues	[92,93,94]
Iron	30–50%	1000 to 1350 nm	Best penetration depth due to operating at NIR-II (1000–1350 nm) Biocompatibility Accumulation in tumors can be enhanced with external magnetic field.	Moderate PCE Optimization with doping to improve PCE NIR-II window increases toxicity risk in skin	[95]
Zirconium	40%	808 nm	Combination with RT	Early stage research Many uncertainties regarding regulation and safety	[96]

ChT: chemotherapy; NIR: near-infrared; NPs: nanoparticles; PCE: photothermal conversion efficiency; RT: radiotherapy.

Consistent with these findings, another approach involves encapsulating doxorubicin within polydopamine NPs, where laser irradiation similarly enables a synergistic PTT–ChT effect. Notably, this strategy may offer broader applicability, as it is not limited to TNBC or other breast cancer subtypes requiring the presence of BRCA1 mutations [97]. This suggests that polydopamine-based PTT platforms may serve as versatile carriers for different ChT agents, extending their potential beyond genetically defined patient populations.

## 3. Light-Based Nanotherapies: Illuminating New Frontiers and Facing Challenges

PDT and PTT each offer distinct advantages over conventional modalities and, in many settings, over each other. In both PDT and PTT, PS/PTAs are primarily retained in tumors, lowering the risk of collateral injury to adjacent healthy tissues [56]. It can also boost the immune response, as apoptosis and regulated necrosis release damage-associated molecular patterns, such as ATP and calreticulin, driving immunogenic cell death and downstream immune activation [98]. Lastly, PDT combines well with ChT, enabling dose reductions and thereby decreasing the systemic side effects [97,99].

By contrast, PTT does not need oxygen to be performed, an advantage in hypoxic tumors where PDT efficacy may be limited [100,101]. In addition, it offers precise spatial–temporal control. Following intravenous administration, nanoparticle selectivity can be further improved using AuNPs through both passive (mainly in animal models) accumulation and active molecular targeting mechanisms [88,102]. Depending on clinical goals, PTT can achieve ablative heating or be applied as mild-PTT (sub-ablative, ~42–45 °C) to sensitize tumors and potentiate combination regimens.

Both modalities can be used as monotherapy or can be combined to engage complementary mechanisms. PTT provides rapid, local cytotoxicity and vascular effects, while PDT supplies ROS-mediated killing and immune priming. Although combination therapy can increase costs and complexity, attacking orthogonal pathways helps circumvent resistance and may allow lower individual doses of tumor [101,103,104,105].

Although light-based therapy does not imply the typical drug used in cancer treatment, it still has a long way to go, with few disadvantages. Something that is still a major challenge to both is the limited light penetration with an external light source, but it can be solved by an interstitial or intracavitary light delivery to place the source closer to the target volume [87]. In case of uncontrolled thermal increment in PTT, it can lead to an increase in the probability of necrosis, leading to local inflammation [106,107]. Careful dosimetry/thermometry and preference for mild-PTT in combinations can reduce this risk. In PDT, the same can be described, although more superficially, where the skin can become photosensitive, hence becoming more susceptible to adverse events [108]. Additionally, PDT has an oxygen dependency to create ROS, as previously mentioned [100,101], and PDT further enhances the hypoxia conditions that are pre-existing in the TME. This may lead to unsuccessful treatment.

**Figure 6:**
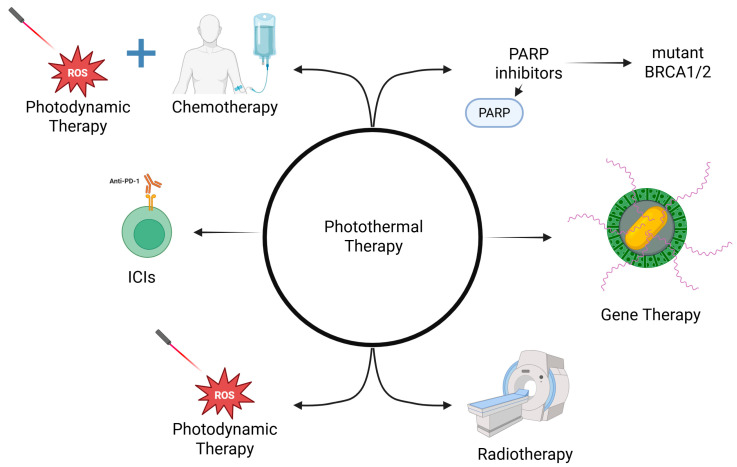
Schematic figure representing a combination of cancer therapies and PTT. BRCA means breast cancer gene; ICI: immune checkpoint inhibitors; MRI: magnetic resonance imaging; ROS: reactive oxygen species. Created with BioRender.com.

On the other hand, PTT is constrained by the thermal sensitivity of living tissues. If heating is insufficient, treatment can be ineffective; if excessive, collateral damage may occur. In addition, tumors can acquire thermotolerance through the overexpression of heat-shock proteins (e.g., HSP70/HSP90), which protect cells from thermal stress and blunt PTT efficacy [109,110]. Mitigation strategies under investigation include mild-PTT combined with chemotherapy, PDT or IT, and the pharmacological inhibition of HSPs, to lower the thermal dose required and curb adaptive resistance. Figure 6 presents combinations of PTT with other treatment modalities.

A further challenge—common to most locoregional therapies—is metastatic disease. PTT cannot, by itself, prevent metastasis; the effective treatment of established metastases generally requires both (i) adequate drug/NP or PS deposition in each lesion and (ii) light delivery to those sites (e.g., image-guided interstitial fibers), which is not always feasible [111]. This reality underpins growing interest in systemic combinations (ChT-/IT) and in deeper-penetrating illumination (e.g., NIR-II) to extend PTT’s reach.

Clinical translation is in progress. Early-stage and pilot trials of NP-assisted light therapies are assessing the safety, effectiveness, and translational potential of these nanoparticle-assisted therapies. Although many programs remain in preclinical or phase I trials, initial data are promising, indicating enhanced tumor regression, reduced side effects, and improved survival rates in selected settings, including TNBC. Even so, larger, controlled trials are needed to validate safety, optimize thermal/optical dosing, and confirm durable benefit.

Several patents have emerged to support the needs of these clinical trials. These new inventions are designed to advance light-based therapies, which are yet to reach human clinical trials. There are several treatment modalities proposed by the patented drugs, such as (1) NP with a prodrug to combine ChT, TT, PDT, and IT or by themselves (Figure 6) [112]; (2) NPs for PTT and also imaging [113,114,115,116,117]; (3) targeting thermal conditions and immune system modulation [118,119,120,121]. Table 4 summarizes some patents that collectively illustrate how nanomaterials—particularly Au, silicon, lipid-based, and hybrid nanoparticles—are being engineered to integrate PTT, PDT, ChT, and IT modalities into unified therapeutic systems.

The earliest examples, such as US 11246877 B2, represent a paradigm shift toward multimodal nanoparticle systems, designed to enhance drug specificity and enable combination therapy within a single platform [112]. Such technologies allow for the precise control of drug release, tumor targeting, and reduced systemic toxicity, making them ideal for aggressive and treatment-resistant cancers like TNBC.

Patents US 20140296836 A1 and US 20150065858 A1 exemplify the theragnostic evolution, which is the merging of diagnostic and therapeutic capabilities within a single nanosystem [113,114]. The incorporation of MRI and photoacoustic imaging enhances the real-time monitoring of tumor response and enables adaptive treatment strategies. Similarly, the gold–silicon hybrid nanostructures demonstrate exceptional photothermal conversion efficiency, allowing precise tumor ablation under NIR light exposure.

Other patents, such as US 20140220143 A1 and US 20240285760 A1, extend this innovation frontier by coupling PTT with immune activation [118,119]. These approaches utilize photoactive hybrid nanoparticles or the thermal modulation of T cells to stimulate antitumor immunity, marking a significant step toward immunophotothermal synergy. Such integration could potentially transform local tumor destruction into systemic immune protection, which is an area of considerable promise in modern oncology.

Notably, US 20240293573 A1 and US 20180133319 A1 highlight the advancement of targeted and synergistic nanotherapies, employing antibody-conjugated lipid nanoparticles or multi-mechanistic nanosystems to optimize selectivity and therapeutic outcomes [120,121]. These technologies signify the maturation of nanomedicine from experimental proof-of-concept to clinically translatable, precision-driven interventions. Collectively, the patents reveal a consistent focus on multifunctionality, combining therapeutic and diagnostic (theragnostic) capabilities; precision and control, through antibody or receptor-based targeting; and the integration of immune modulation, expanding the scope of light-based therapies and translation potential, emphasizing biocompatibility and scalability.

The last major issue is the dimensions of a nanoparticle, since the size of NPs is not universally defined. EMA defines a nanoparticle from 1 to 100 nm, although many formulations surpass that scale [122,123]. Therefore, the nanoproducts fall under quite ambiguous categories.

Regarding the regulation of the active pharmaceutical ingredient, NPs can encapsulate the drug, thereby needing to have approval for the pharmaceutical drug encapsulated and for the clearance of the encapsulation material. When thinking of AuNPs for PTT, the dual approval is now for the NP and for the laser system employed. Hence, due to the lack of FDA and EMA consensus and protocols regulating the physicochemical characteristics of the nanomaterials, it is expected that each research group determines the critical parameters that ensure both quality and safety [124]. Lastly, the lack of human clinical trials does not allow any in-depth knowledge regarding toxicity concerns.

## 4. Conclusions

TNBC remains one of the most aggressive and therapeutically challenging BC subtypes. Despite incremental progress with ChT, TT, and IT, patient outcomes remain suboptimal due to tumor heterogeneity and the absence of actionable molecular targets. This underscores the urgent need for innovative therapeutic modalities that can bypass resistance mechanisms, selectively target tumor cells, and minimize systemic toxicity.

In this context, light-based therapies like PDT and PTT are being actively explored, particularly within the framework of nanotechnology. While their clinical effectiveness remains to be fully validated, extensive preclinical studies in both in vitro and in vivo models have demonstrated encouraging results.

Among these, PTT combined with AuNPs has gained significant attention due to its superior photothermal conversion efficiency, biocompatibility, and ability to induce localized tumor ablation. Moving forward, the translation of these preclinical findings into well-designed clinical trials will be essential to evaluate their safety, pharmacokinetics, and therapeutic performance in human patients. Looking ahead, the critical next step is the translation of these findings into clinical trials to assess safety and efficacy in human patients. With continued research, there is hope that PTT—especially in combination with nanotechnology—may emerge as a viable and effective therapeutic option for TNBC.

## Figures and Tables

**Table 1:** Standard-of-care treatment for TNBC, according to ESMO guidelines.

Stage	Key Biomarkers/Mutation Testing	First-Line Therapy	Additional Treatments	Adjuvant Therapy
Early-stage/localized (Stage I)	ER/PR/HER2 negativity	Surgery	Whole breast RT as indicated by surgical margins.	Consider systemic ChT if nodular invasion; if gBRCA1/2 mutation-positive and add olaparib for 1 year.
	Germline BRCA 1/2 testing			
Early-stage/localized (Stage II-III)	ER/PR/HER2 negativity	Neoadjuvant ChT (anthracycline + taxane; add platinum in high-risk cases) plus pembrolizumab; followed by surgery.	Locoregional RT as indicated by surgical margins.	If pCR achieved: pembrolizumab. If residual disease: continue pembrolizumab. Consider capecitabine; If gBRCA1/2 mutation-positive add olaparib for 1 year.
	Germline BRCA 1/2 testing			
	PD-L1 for trial eligibility			
Metastatic PD-L1 positive	PD-L1 expression	IT + ChT (Atezolizumab or Pembrolizumab + nab-paclitaxel)	Sacituzumab govitecan Or ChT (eribulin, capecitabine or vinorelbine)	-
	ER/PR/HER2 negativity in recurrence			
Metastatic PD-L1 negative and gBRCA-mt	BRCA1/2 mutations	PARP inhibitor	-	-
Metastatic PD-L1 negative and gBRCA-wt	NO BRCA1/2 mutations	Taxane/Anthracycline monotherapy.	Imminent organ failure: Bevacizumab + Taxane/Capecitabine Or Anthracycline + Taxane Combination	-
Early relapse (≤6–12 months after neoadjuvant/adjuvant ICI)	BRCA1/2 mutations	gBRCA mutation: PARP-inhibitor Or ChT gBRCA-wt: Sacituzumab govitecan Or Cht	Progression: Sacituzumab govitecan (if not used already) Or Trastuzumab deruxtecan Or ChT (eribulin, capecitabine or vinorelbine)	-

BRCA: Breast cancer gene; ChT: chemotherapy; ER: estrogen receptor; gBRCA: germline BRCA; HER2: human epidermal growth factor receptor 2; ICI: immune checkpoint inhibitor; IT: immunotherapy; PARP: poly ADP ribose polymerase-1; pCR: pathological complete response; PD-L1: programmed death ligand 1; PR: progesterone receptor; RT: radiotherapy.

**Table 2:** AuNP characteristics and their impact on PTT efficiency in TNBC.

Properties	Impact in PTT Efficiency	References
Size	<20 nm: Better conversion efficiency, but lower absolute heat. Absorption at UV (540 nm). Risk of unspecific systemic distribution to various organs.	[68,72]
	20–100 nm: Stable, achieve worse heat conversion efficiency, although higher absolute heat compared to smaller ones. Passive targeting and EPR effect. Tendency to aggregate. Preferential if IV administration	
	100–200 nm: Biocompatible, large enough either to avoid the passive transport and small enough to avoid the RES. Decreased filtration from liver and spleen. Increased probability of clearance by monocytes in IV perfusion. Preferential if administration is in situ.	
	>200 nm: Tend to accumulate in organs of the reticuloendothelial system. Increased filtration from liver and spleen	
Morphology	Nanospheres: Lower photothermal efficiency despite greater stability and biocompatibility. Uniformity	[72,73,74,75]
	Nanorods: Possible adjustment due to variable shape. Photothermal efficiency is decreased due to high polydispersity conditions, leading to variable shapes and dimensions. Surfactant is employed in the synthesis, which can be a concern regarding toxicity.	
	Nanoshells: Tunable NIR region absorption band, due to modifying the core/shell ratio. Core can be either a dielectric material or a drug encapsulated.	
	Nanostars: Enhanced photothermal efficiency when compared to spheres and rods.	
Surface Modification Strategies	PEGylation: Vastly increases AuNPs half-life, since PEG protects from protein adsorption and liver uptake. Targeting ligands, such as folate, with the respective receptor being overexpressed in TNBC.	[73,75,76]

AuNPs: gold nanoparticles; EPR: enhanced permeability retention; IV: intravenous; PTT: photothermal therapy; TNBC: triple-negative breast cancer; UV: ultraviolet.

**Table 4:** Recent patents in light-based and combination therapies.

Patent Number	Patent Title	Therapeutic Category	Key Features	Potential Clinical Application	Reference
US 11246877 B2	NPs for ChT, TT, PDT, IT, and Combinations Thereof	Multimodal Therapy	Multifunctional NPs for ChT, TT, and IT delivery with PDT activation.	Broad-spectrum oncological use; combinatorial nanotherapy for resistant cancers.	[112]
US 20140296836 A1	Gold-in-Silicon Nanoassembly for Thermal Therapy and Methods of Use	PTT	Hybrid gold–silicon nanostructures with high photothermal conversion efficiency for local tumor ablation via NIR irradiation.	Localized PTT in solid tumors (e.g., BC, prostate).	[113]
US 20150065858 A1	Core–Satellite Nanocomposites for MRI and PTT	Theragnostic	Dual-function nanocomposites integrating imaging (MRI) and PTT effects, enhancing diagnosis–therapy integration.	Image-guided PTT cancer therapy.	[114]
US 20250034158 A1	Texaphyrin Derivatives for Manganese ChT, Photoacoustic Imaging, and PTT.	Combined Therapy and Imaging	Texaphyrin-based agents combining chemotherapy, PTT, and photoacoustic imaging for tumor targeting.	Diagnostic and therapeutic tool for multi-resistant tumors.	[115]
US 20200384110 A1	Biocompatible PTT Compositions for Cancer and Skin Diseases	PTT	Biocompatible structures that target the tumor sites and can be used for PTT.	Non-invasive cancer and dermatological therapy.	[116]
US 20250136629 A1	Self-Assembled NPs for PTT	PTT	Fe NPs used in PTT to work around the issue of low PT conversion.	PTT for tumors.	[117]
US 20140220143 A1	Immune-Stimulating Photoactive Hybrid Nanoparticles	PTT + IT	Hybrid nanoparticles inducing photothermal tumor lysis while enhancing immune cell activation.	IT-PTT for metastatic tumors.	[118]
US 20240285760 A1	Methods and Compositions for Remote Control of T Cell Therapies via Thermal Targeting	IT + PTT	Remote thermal modulation to guide and enhance T-cell therapies using external heat stimuli.	Controlled activation of engineered T-cells in solid tumors.	[119]
US 20240293573 A1	Pharmaceutical Composition for Treating Cancer Lipid–Photothermal NPs Conjugated with Antibodies	TT + PTT	Lipid-based PTT NPs functionalized with antibodies for precise tumor targeting.	Antibody-directed PTT for breast and ovarian cancers.	[120]
US 20180133319 A1	Synergistic Nanotherapy Systems and Methods of Use Thereof	Combination Therapy	Multifunctional nanoplatforms integrating ChT, photo-, and IT effects.	Personalized and adaptive cancer therapy systems.	[121]

ChT: chemotherapy; IT: immunotherapy; MRI: magnetic resonance imaging; NPs: nanoparticles; PDT: photodynamic therapy; PTT: photothermal therapy; TT: targeted therapy.

## Data Availability

No new data were created or analyzed in this study.

## Abbreviations

The following abbreviations are used in this manuscript:

ADC	Antibody-Drug Conjugates
A375	Melanoma cell line
AuNPs	Gold Nanoparticles
BC	Breast Cancer
B16F10	Murine melanoma cell line
BRCA	Breast cancer gene
CAR-T	Chimeric Antigen Receptor T-cell
ChT	Chemotherapy
CTLA-4	Cytotoxic T-Lymphocyte Antigen-4
EGFR	Epidermal Growth Factor Receptor
EPR	Enhanced Permeability Retention
ER	Estrogen Receptor
ESMO	European Society for Medical Oncology
FDA	U.S. Food and Drug Administration
GV	Glembatumumab vedotin
HaCaT	Human keratinocytes
HAOA	Hyaluronic Acid-Oleic Acid
HCT-116	Colon cancer cell line
HER-2	Human Epidermal Growth Factor Receptor-2
ICI	Immune Checkpoint Inhibitors
IV	Intravenous
IT	Immunotherapy
JAK	Janus Kinase
MCF-7	BC cell line
MRI	Magnetic Resonance Imaging
N#	Nodule invasion
NK cell	Natural-killer cell
NIR	Near-infrared
NP	Nanoparticle
PDT	Photodynamic Therapy
PD-L1	Programmed Death Ligand-1
PI3K	Phosphoinositide 3-Kinase
PR	Progesterone Receptor
PARP1	Poly ADP Ribose Polymerase-1
PCE	Photothermal Conversion Efficiency
PS	Photosensitizer
PTT	Photothermal Therapy
ROS	Reactive oxygen species
RT	Radiotherapy
SG	Sacituzumab govitecan
T#	Tumor staging
TME	Tumor Microenvironment
TNBC	Triple-negative Breast Cancer
TT	Targeted Therapy
UV	Ultraviolet
VEGFR	Vascular Endothelial Growth Factor Receptor
